# Unveiling a novel transient druggable pocket in BACE-1 through molecular simulations: Conformational analysis and binding mode of multisite inhibitors

**DOI:** 10.1371/journal.pone.0177683

**Published:** 2017-05-15

**Authors:** Ornella Di Pietro, Jordi Juárez-Jiménez, Diego Muñoz-Torrero, Charles A. Laughton, F. Javier Luque

**Affiliations:** 1 Laboratory of Pharmaceutical Chemistry, Faculty of Pharmacy and Food Sciences, and Institute of Biomedicine, University of Barcelona, Barcelona, Spain; 2 Department of Nutrition, Food Science and Gastronomy, Faculty of Pharmacy and Food Sciences, and Institute of Biomedicine, Campus Torribera, University of Barcelona, Santa Coloma de Gramenet, Spain; 3 School of Pharmacy and Centre for Biomolecular Sciences, University Park, Nottingham, United Kingdom; Bioinformatics Institute, SINGAPORE

## Abstract

The critical role of BACE-1 in the formation of neurotoxic ß-amyloid peptides in the brain makes it an attractive target for an efficacious treatment of Alzheimer’s disease. However, the development of clinically useful BACE-1 inhibitors has proven to be extremely challenging. In this study we examine the binding mode of a novel potent inhibitor (compound **1**, with IC_50_ 80 nM) designed by synergistic combination of two fragments—huprine and rhein—that individually are endowed with very low activity against BACE-1. Examination of crystal structures reveals no appropriate binding site large enough to accommodate **1**. Therefore we have examined the conformational flexibility of BACE-1 through extended molecular dynamics simulations, paying attention to the highly flexible region shaped by loops 8–14, 154–169 and 307–318. The analysis of the protein dynamics, together with studies of pocket druggability, has allowed us to detect the transient formation of a secondary binding site, which contains Arg307 as a key residue for the interaction with small molecules, at the edge of the catalytic cleft. The formation of this druggable “floppy” pocket would enable the binding of multisite inhibitors targeting both catalytic and secondary sites. Molecular dynamics simulations of BACE-1 bound to huprine-rhein hybrid compounds support the feasibility of this hypothesis. The results provide a basis to explain the high inhibitory potency of the two enantiomeric forms of **1**, together with the large dependence on the length of the oligomethylenic linker. Furthermore, the multisite hypothesis has allowed us to rationalize the inhibitory potency of a series of tacrine-chromene hybrid compounds, specifically regarding the apparent lack of sensitivity of the inhibition constant to the chemical modifications introduced in the chromene unit. Overall, these findings pave the way for the exploration of novel functionalities in the design of optimized BACE-1 multisite inhibitors.

## Introduction

BACE-1 (also known as ß-secretase; EC 3.4.23.46) is a membrane-associated, pepsin-like aspartic protease responsible for the cleavage of the amyloid precursor protein (APP), which gives rise to toxic ß-amyloid (Aß) peptides of various lengths, including the most pathogenic isoform, Aß42, one of the main hallmarks of Alzheimer’s disease (AD) [1]. The early formation of Aß in the AD neurotoxic pathogenic cascade and the slowed memory decline found upon abolished Aβ production in BACE-1 knockout mice [2–5] suggest that BACE-1 may be a prime biological target for a disease-modifying therapeutic approach in AD [6–8].

The more than 230 crystal structures so far deposited in the Protein Data Bank (PDB) [9] attest that the BACE-1 active site is an open, long cleft formed between the N- and C-terminal lobes. Encompassing a volume close to 1000 Å^3^, this cleft is formed around two catalytic Asp residues, Asp32 and Asp228, which face each other generally in a non-coplanar orientation [10,11]. The binding site cleft is partially covered by a highly flexible antiparallel hairpin-loop, known as the *flap*. The open conformation of the flap in apo structures of the enzyme [12–14] would allow the access of the substrate and the release of hydrolysis products. In contrast, a closed conformation is found in ligand-bound complexes, even though the degree of opening/closure varies among different X-ray structures [15–17], thus reflecting the structural flexibility of the flap.

Despite the huge research effort invested to gain insight into a number of issues such as the substrate specificity, the protonation state of the catalytic dyad, the presence of allosteric sites, and the enzyme’s functional plasticity [18–20], the impact on the design of novel drugs has been limited, even though ongoing efforts have led to the exploration of novel BACE inhibitors, such as LY3202626, CNP520 and JNJ-54861911, in clinical trials [21]. The huge size of the enzyme active site cleft and the proper balance of pharmacokinetic and pharmacodynamic properties of the inhibitors, including good oral bioavailability and brain penetration as well as selectivity against BACE-1 relative to other aspartyl proteases, have challenged the development of clinically useful BACE-1 inhibitors. Many endeavours have been focused on achieving high affinity and selectivity by exploiting the different subsites that are present in the catalytic cleft, while modulating the pharmacokinetic properties, blood-brain barrier penetration, and susceptibility to P-glycoprotein-mediated efflux [22–25]. Generally, BACE-1 inhibitors have been designed to target eight subsites (S_1_-S_4_ and S_1_’-S_4_’; S1 Fig) [26], though there have been attempts to exploit additional subsites extending beyond S4 [27,28].

**Fig 1:**
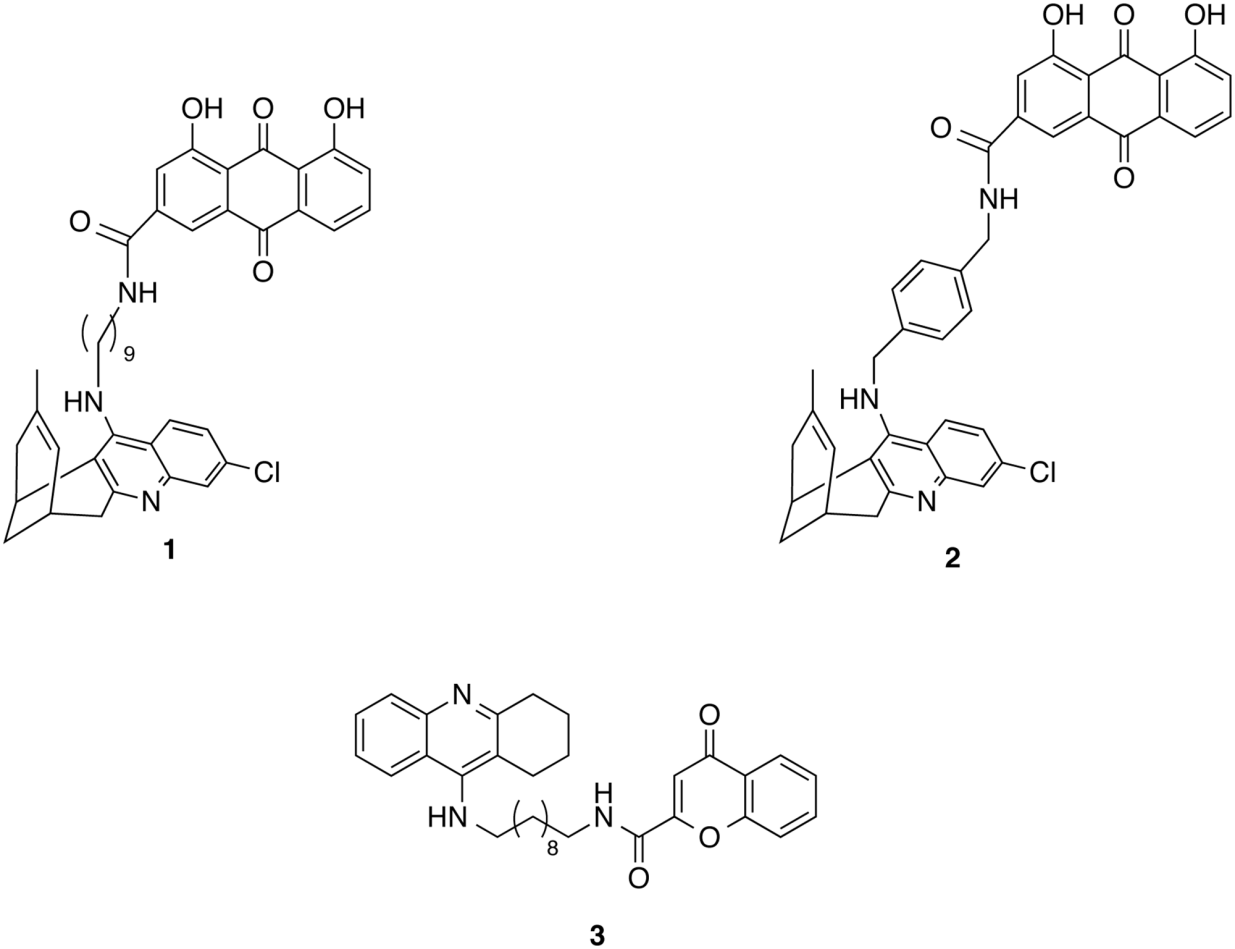
Chemical structures of the huprine-rhein hybrids 1 and 2 and of the 6-chlorotacrine-chromene hybrid 3.

We have recently reported the outstanding *in vitro* and *in vivo* pharmacological profile displayed by the enantiomeric forms of the novel rhein-huprine hybrid compound **1** (Fig 1) as multitarget disease-modifying anti-Alzheimer agents. The two enantiomers were unexpectedly endowed with a remarkable inhibitory potency against BACE-1 (IC_50_ = 80 nM) [29], even though the fragment-based linking strategy was originally designed without targeting specifically BACE-1. Indeed, no significant inhibitory activity was found for the two model compounds from which hybrid **1** had been designed, huprine Y and rhein. Thus, the inhibitory potency of huprine Y in BACE-1 was found to be 13.6 ± 2.3% and 14.0 ± 0.1% at 5 μM for the (+)-(7*R*,11*R*)- and (-)-(7*S*,11*S*)-enantiomer [30], respectively, while no inhibition activity was found for rhein at this concentration [29]. This suggests that the precise spatial arrangement in the BACE-1 cleft afforded by tethering of huprine and rhein triggered a highly synergistic cooperative effect, which is reflected in a >100-fold enhancement in binding affinity. Therefore, we hypothesize that the potent inhibitory activity of compound **1** originates from a multisite binding. This would justify the marked dependence of the inhibitory activity on the length of the methylenic tether chain that was linking huprine and rhein moieties, with the nonamethylene tether in hybrid **1** providing the optimal distance for a cooperative binding to BACE-1.

Previous computational studies on a much less potent member of the same structural class, **2** (IC_50_ = 2020 nM; Fig 1), predicted that the protonated aminoquinoline ring of the huprine moiety interacts with the catalytic dyad, while forming multiple hydrophobic contacts between the heterocyclic ring and residues Leu30, Phe108, Ile110, and Ile118 [29]. Furthermore, the strong dependency of the inhibitory potency on the tether length prompted us to hypothesize that the rhein moiety of **1** was capable of filling a peripheral “floppy” pocket in the region delimited by the highly flexible loops formed by residues 8–14, 154–169, and 307–318.

In addition, the low micromolar *K*_*i*_ values against BACE-1 in independent studies by Fernández-Bachiller *et al*. [31] for a series of structurally related decamethylene-linked tacrine-chromene hybrids seemed attainable through adoption of a similar multisite binding to both catalytic and peripheral sites. Thus, while it is reasonable to expect that the tacrine moiety of the tacrine-chromene hybrids will bind to the catalytic dyad, mimicking the binding of the huprine unit in the huprine-rhein hybrids, it might be speculated that both rhein and chromene units could bind the same secondary pocket in the enzyme.

In this study we have devised a multistep computational protocol to explore the conformational flexibility of the enzyme catalytic cleft in order to identify the nature and binding features of the “secondary” site that presumably accommodates both rhein and chromene moieties in hybrids **1** and **3**. In particular, in this study we report the results of the conformational analysis and clustering studies of the flexible region defined by loops 8–14, 154–169, and 307–318 in BACE-1. The conformational flexibility of this region in the apo form of the enzyme was studied by means of extended molecular dynamics (MD) simulations and the conformational preferences were determined in order to identify the formation of a secondary pocket in this highly flexible region. The druggability analysis of the transient pocket formed in this region supported the feasibility to bind the rhein/chromene moieties. Finally, the structural integrity of the dual site binding of huprine-rhein and tacrine-chromene hybrids **1** and **3**, filling both catalytic and secondary pockets, was explored by MD simulations.

## Methods

**Fig 2:**
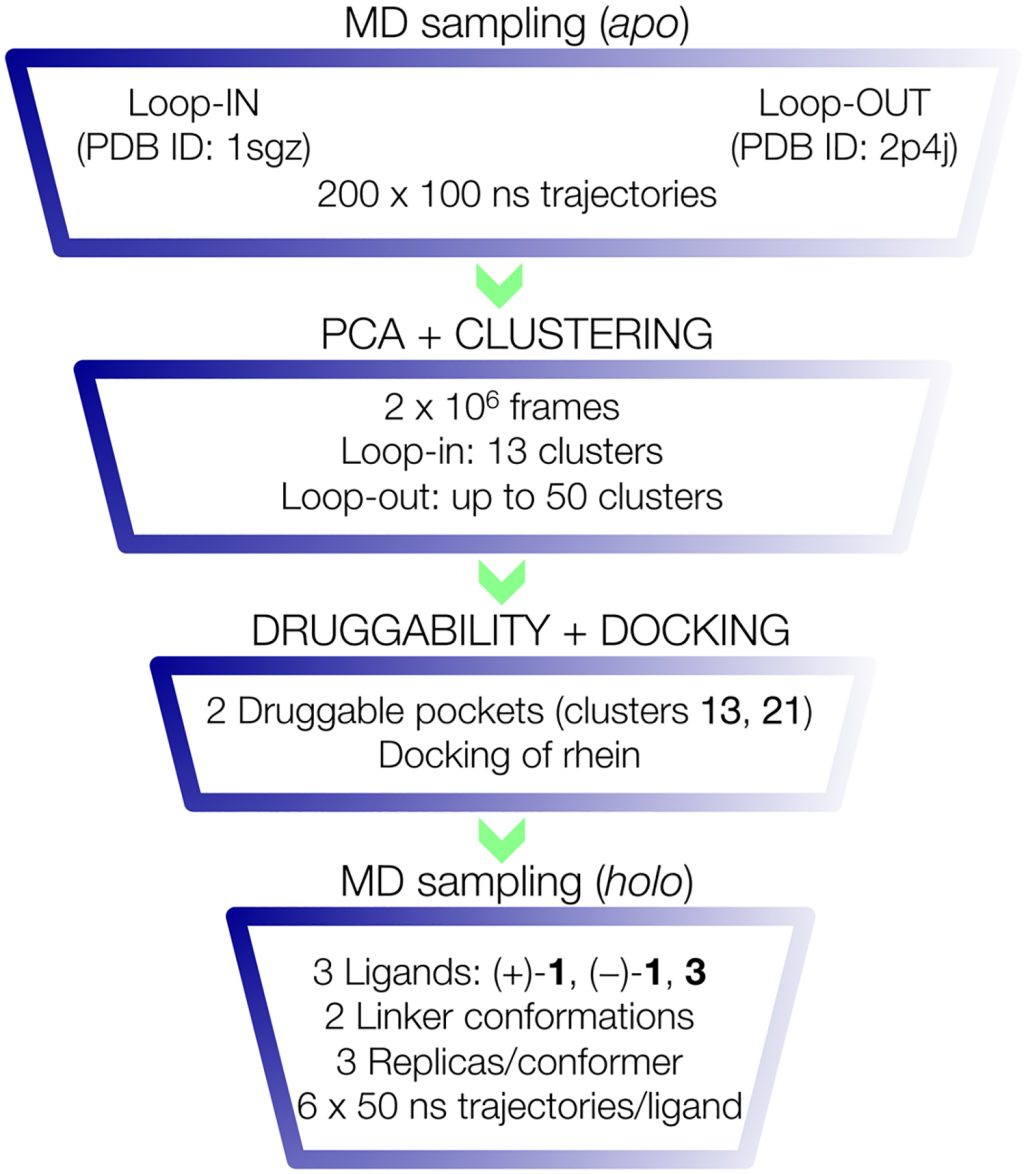
Representation of the multistep computational approach utilized in the focusing search strategy followed in this study.

Because the region defined by loops 8–14, 154–169, and 307–318 in BACE-1 appears to be highly flexible, we hypothesize that the cooperative binding between huprine and rhein moieties in compound **1** is assisted by the transient formation of a druggable pocket. Nevertheless, inspection of the BACE-1 X-ray structures deposited in the PDB failed in finding a well-shaped pocket in this region that could satisfy the requirements for binding of the rhein unit and the length of the tether between huprine and rhein moieties in hybrid **1**. Accordingly, we devised the computational protocol shown in Fig 2, which was designed as a focusing strategy leading from i) an exhaustive sampling of the conformational space of the terminal region of the BACE-1 catalytic cleft to ii) the identification of a “floppy” pocket by combining principal component analysis of the sampled structures of the apo enzyme, iii) clustering of the major conformational families, and iv) druggability analysis of the putative pockets. Finally, selection of the best pose of rhein/chromene units was accomplished by docking calculations, and the structural integrity of the dual site binding mode of the hybrid compounds was carried out through the analysis of MD simulations. A summary of the computations can be found in S1 Table, and details of the different computational approaches in the focusing search strategy are given below.

### Setup of the molecular system

Computational studies were performed using the X-ray crystallographic structures of human BACE-1 in the apo (PDB ID 1SGZ) [12] and ligand-bound forms (PDB ID 2P4J) [32]. Choice of these structures was motivated by two major reasons. First, whereas the loops 8–14, 154–169, and 307–318, which shape a highly flexible region at the end of the catalytic cleft, are not visible in most of the X-ray structures, structures 1SGZ and 2P4J are among the few ones that display the spatial arrangement of these loops. Second, the analysis of the two X-ray structures reveals two distinct conformations for the loop formed by residues 8–14, which also affect the spatial arrangement of Arg307. Hereafter these conformations will be denoted *loop-in* (1SGZ) and *loop-out* (2P4J), as indicated in Fig 2. Accordingly, they were chosen as templates to generate the starting systems for conformational sampling through extended MD simulations.

The structures of the simulated systems were refined by removal of the complexed ligand in 2P4J and addition of missing hydrogen atoms. Furthermore, acetyl and *N*-methyl groups were added to the N-terminal and C-terminal residues, respectively. The ionization state for the rest of ionizable residues was assessed through PROPKA3.0 calculations [33,34], and checked for consistency with the pH used in experimental assays (pH of 4.5; [29]) upon visualization of specific environment of ionizable residues in the X-ray structures. Protonation of histidines is expected to have minor effects on the protein dynamics due to the large solvent exposure of these residues. In contrast, protonation of the catalytic dyad is more relevant for ligand binding, as has been discussed elsewhere [21]. Specifically, simulations were performed in the monoprotonated state, with neutral Asp228 and deprotonated Asp32. In addition, three disulfide bridges were defined between Cys residues 155–359, 217–382, and 269–319. Structural waters were retrieved from those found in the X-ray structures, paying attention to the water molecules along the catalytic cleft.

### Molecular dynamics simulations of the apo enzyme

The conformational space of the apo form of BACE-1 was explored by means of extended MD simulations. To this end, each of the two structural models generated from structures 1SGZ and 2P4J was sampled by means of 100 independent MD simulations, each covering 100 ns. This strategy was motivated by a twofold reason. First, the need to explore exhaustively the conformational space defined by the three flexible loops that shape the secondary pocket, keeping in mind the lack of structural data about these loops in most X-ray structures. Second, to assess the convergence of the samplings started from *in* and *out* conformational states. In fact, this was the major concern, as we wanted to be sure that simulations starting from the *in* (*out*) conformational state were able to visit the other arrangement, thus giving confidence to the whole conformational sampling.

Calculations were performed using GROMACS 4.7 software package [35]. Parameters for the protein were taken from the parm99SB [36] force field. Na^+^ cations were added to neutralize the negative charge of the system. The simulated systems were immersed in a cubic box of TIP3P water molecules [37], preserving the crystallographic waters inside the binding cavity. Each of the two final systems contained around 45,000 atoms.

The geometry of the system was minimized through 1,000 steps of steepest descent algorithm, with a step size of 0.001 nm and a tolerance of 250 kJ·mol^-1^·nm^-1^. Thermalization was accomplished in four steps. During the first three steps (300 ps), all bonds were constrained and temperature was gradually increased to 300 K. Then, a 10 ns MD was performed in order to equilibrate the density according to the Parrinello-Rahman extended-ensemble pressure coupling. At this point, 100 independent replicas for each of the two systems were generated by randomly assigning different sets of velocities (adjusted to a temperature of 300 K) to the initial coordinates. For each replica, a 100 ns trajectory was run using a time step of 2 fs in conjunction with bond length constraints using LINCS [38], periodic boundary conditions at constant pressure (1 bar) and temperature (300 K, using the velocity-rescaling temperature coupling algortihm), particle mesh Ewald [39,40] for the treatment of long electrostatic interactions. A cutoff of 10 Å was utilized for nonbonded interactions.

### Conformational flexibility

Principal Component Analysis (PCA) [41] was used to examine the conformational variability in BACE-1 using a locally modified version of pyPCAzip package [42]. The analysis was focused on the region shaped by loops 8–14, 154–176, and 307–318 (including 484 heavy atoms). To this end, a total of 1,000,000 frames were extracted from each simulation of the apo enzyme. These frames were evenly taken over the last 50 ns of the trajectories. PCA was also performed for a set of 17,080 frames composed of apo and holo structures. The pyPCAzip package was also used for the clustering of the protein conformational ensemble, based on watershed partitioning of the free energy surface created by Boltzmann inversion of the probability density map in the PC1/PC2 subspace.

### Druggability predictions

The Epock program [43] was used to characterize the cavity volume. Calculations were run on selected protein structures representative of open and closed conformations of the *apo* enzyme, and the *holo* conformation of the pocket. Parameters for grid generation were set as follows. Through the VMD plugin, an including-sphere of 6.0 Å radius, centered in the center-of-mass of the pocket, and several excluding-spheres were used to define the maximum region where the pocket lies. Grid-spacing was set to the default value (0.5 Å). A contiguous-cutoff parameter, which defines the “free space points” that lie within the grid space and distant from any atom less than the cutoff value itself, was also set as default to 4 Å. The fPocket program [44] was used for binding pocket detection and characterization of druggability, which is estimated by combining residue-based descriptors of local hydrophobicity and polarity [45]. A list of the PDB structures generated by clusterization of the whole BACE-1 apo conformational ensemble was provided as input file to fPocket analysis. Parameters were set to the default values.

### Docking calculations

On the basis of the topological and druggability features of the selected clusters, the suitability to accommodate the rhein unit was explored by docking calculations performed with the rDock program [46]. To this end, a cavity of radius 6 Å, centred on the structure of a superligand, containing rhein and an acetamide side chain, was defined as the docking volume. Calculations were performed with no structural waters. 100 Docking poses were generated and sorted according to their score value. The top 50 best scored poses were further analysed by visual inspection.

### Molecular dynamics simulations of the holo enzyme

MD simulations of BACE-1 bound to (+)-(7*R*,11*R*)-**1**, (−)-(7*S*,11*S*)-**1** and **3** were run using AMBER12 software package [47]. The parm99SB force field [35] was used to assign parameters for the protein, while the ligands were parameterized using the GAFF [48] force field in conjunction with RESP charges [49] determined at the B3LYP/6-31G(d) level. For each compound, the amide group attached to the rhein/chromene unit was positioned in two different arrangements, so that the carbonyl group was oriented toward Arg307 or in the opposite way. For each orientation, three distinct replicas slightly differing in the alignment of the oligomethylenic tether along the cleft were chosen. Thus, a total of six independent MD simulations were run for (+)-**1**, (–)-**1**, and **3**.

The geometry of the system was minimized in four steps. First, water molecules were refined through 3,000 steepest descent algorithm followed by 7,000 steps of conjugated gradient. Then, protein and ligand hydrogen atoms positions were optimized using 500 steps of steepest descent and 4,500 of conjugated gradient. Next, the ligand, water molecules, and counterions were further optimized with 2,500 steps of descent and 11,500 of conjugated gradient and, at the last step, the whole system was optimized with 2,500 steps of steepest descent and 8,500 of conjugate gradient. Thermalization was performed in five steps of 25 ps, increasing the temperature from 50 to 300 K. Concomitantly, the residues that define the binding site were restrained during thermalization using a variable restraining force. Thus, a force constant of 25 kcal mol^-1^ Å^-2^ was used in the first stage of the thermalization and was subsequently decreased by increments of 5 kcal mol^-1^ Å^-2^ in the next stages. Then, prior to production simulation, a 10 ns simulation in the isothermal (300 K)—isobaric (1 bar) ensemble was performed in order to reach a stable density. A suitable restraint was used to keep the distance between the carbonyl oxygen atom of rhein and the guanidine moiety of Arg307. Production runs consisted of 50 ns trajectories (accounting for a global simulation time of 300 ns for each ligand) using SHAKE [50] for bonds involving hydrogen atoms, allowing for a time step of 2 fs, in conjunction with periodic boundary conditions at constant pressure and temperature, particle mesh Ewald for long-range electrostatic interactions, and a cutoff of 10 Å for nonbonded interactions.

## Results and discussion

### Analysis of X-ray structures in the PDB

**Fig 3:**
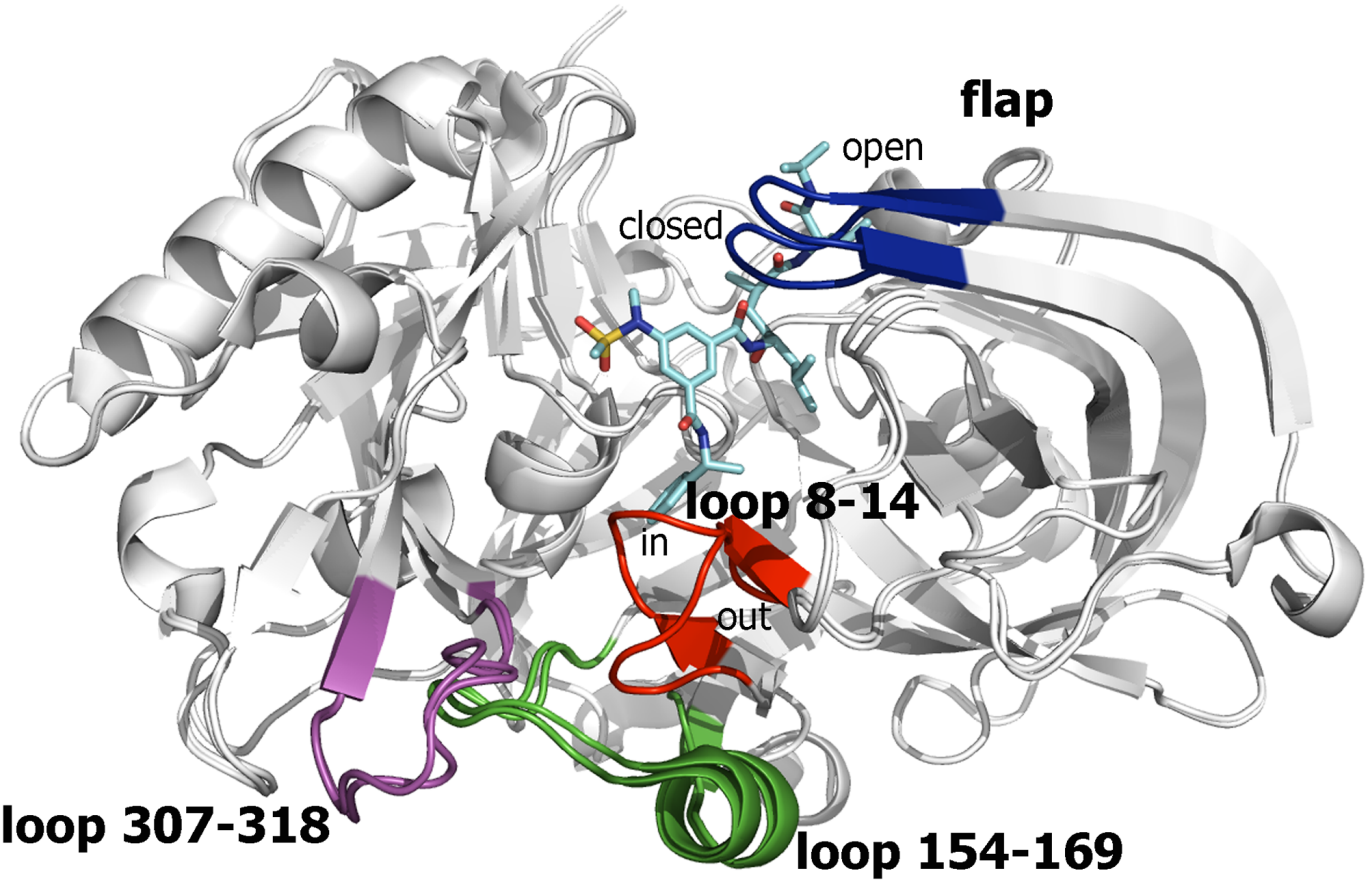
Representation of BACE-1 as found in the structures 1SGZ and 2P4J. The backbone of the superposed proteins is shown as white cartoon, and the ligand located along the catalytic cleft in 2P4J is shown as cyan-colored sticks. The flap region (blue) shows the *open* and *closed* conformations typically found in *apo* and substrate-bound states, respectively. The other flexible region is located in a region distal from the catalytic site and is shaped by residues 8–14 (red), 154–169 (green), and 307–318 (magenta). The two major conformations found for the loop formed by residues 8–14 is shown. Loops 154–169 and 307–318 are generally disordered in other PDB structures.

Inspection of the available X-ray structures of BACE-1 shows that the spatial arrangement of the protein skeleton is well preserved, while conformational flexibility is primarily confined to two regions: i) the “flap” at the binding site, and ii) the loops located at the N-terminal side of the catalytic cleft, including residues 8–14, 154–169, and 307–318 (Fig 3). The loop 8–14 (also known as 10s) can be found in at least two conformations (denoted *in* and *out* in Fig 3), and the transition between these conformations seems to be induced by the specific structural motifs of the ligand [51–53], such as the benzene ring in the inhibitor found in 2P4J. Even though the conformational differences in the loops 154–169 and 307–318 are less apparent in Fig 3, the spatial arrangement of these stretches is not solved in a significant number of the X-ray structures deposited in the PDB, which also suggests that these loops possess a high degree of conformational flexibility.

Further evidence of the flexibility of loops 154–169 and 307–318 comes from recent findings about the allosteric modulation played by antibodies targeting BACE-1 [54,55]. In particular, the noncompetitive mode of inhibition of these antibodies has been attributed to the interaction with an exosite located on the C-terminal lobe, close to the S6 and S7 subsites, shaped by residues 254–257, 270–274, and 309–320. Importantly, such an interaction not only alters the dynamic properties of the covered loops, but even affects the helix spanning residues 157–170 typically present in ligand-bound complexes, which becomes disordered upon antibody binding [19,56].

### Conformational sampling

**Fig 4:**
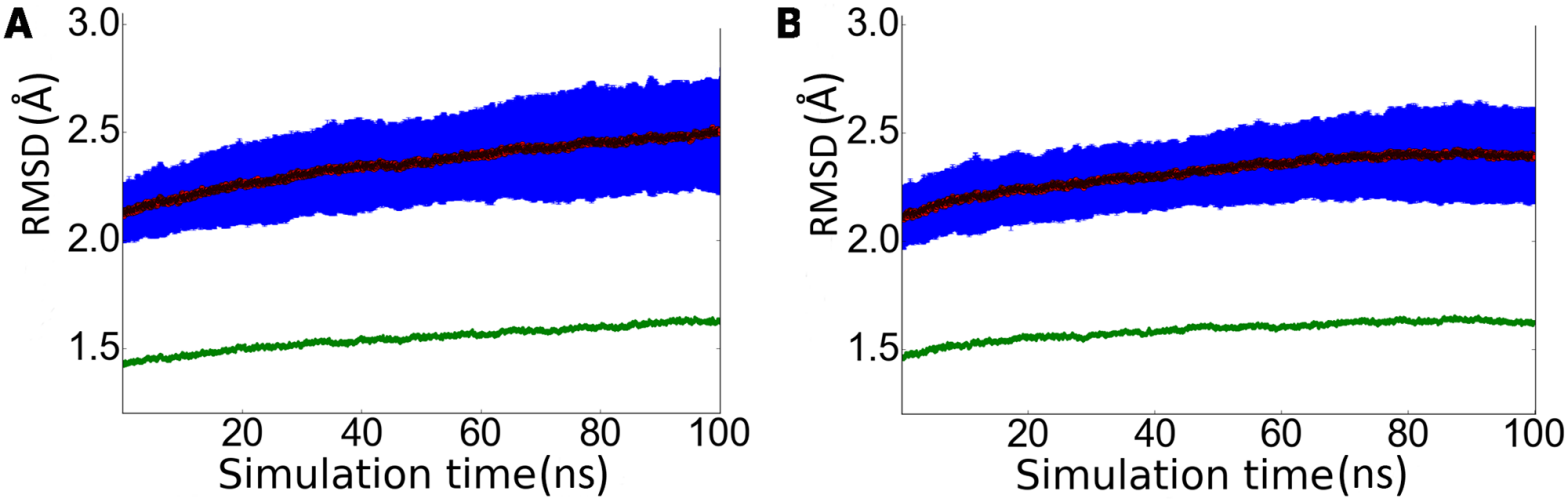
RMSD profile for the ensemble of 100 trajectories started from X-ray structures (A) 1SGZ and (B) 2P4J. The dark blue line reflects the average value of the RMSD (Å) determined for the C-alpha atoms of the whole protein for the ensemble of snapshots collected at a given time (ns) relative to the energy-minimized X-ray structures (the light blue line denotes the variance of the averaged RMSD). The green line reflects the RMSD (Å) determined for the C-alpha atoms of the average structure obtained from the snapshots sampled at a given time (ns) relative to the X-ray structure.

In light of the preceding findings, it can be hypothesized that the conformational flexibility of the loops may facilitate the formation of transient cavities well suited to accommodate the rhein unit present in the hybrid compound **1**. In order to check the feasibility of this hypothesis, extended MD simulations were performed to sample the conformational preferences of loops 8–14, 154–169, and 307–318 from the analysis of 200 independent 100 ns MD simulations started from PDB structures 1SGZ and 2P4J, which were chosen as representative members of the loop 8–14 in both *in* and *out* conformations, respectively. The sampling was deemed sufficient to cover the conformational space of the three loops, as noted in Fig 4, which shows the time evolution of the root-mean square deviation (RMSD) for the ensemble of independent trajectories. Thus, the RMSD profile determined for the whole protein backbone shows a slight increase during the first 80 ns of the whole trajectories, and the achievement of a plateau for the last 10 ns of MD simulations. Furthermore, the overall structure of the protein was well preserved, as noted in the fact that almost 91% of the residues found in helical and β-sheet structures in X-ray structures 1SGZ and 2P4J were retained. The most significant change was the loss of the helical structure formed by residues 154–169, as found in the X-ray structures, an event that contributed to the formation of the secondary site (see below).

**Fig 5:**
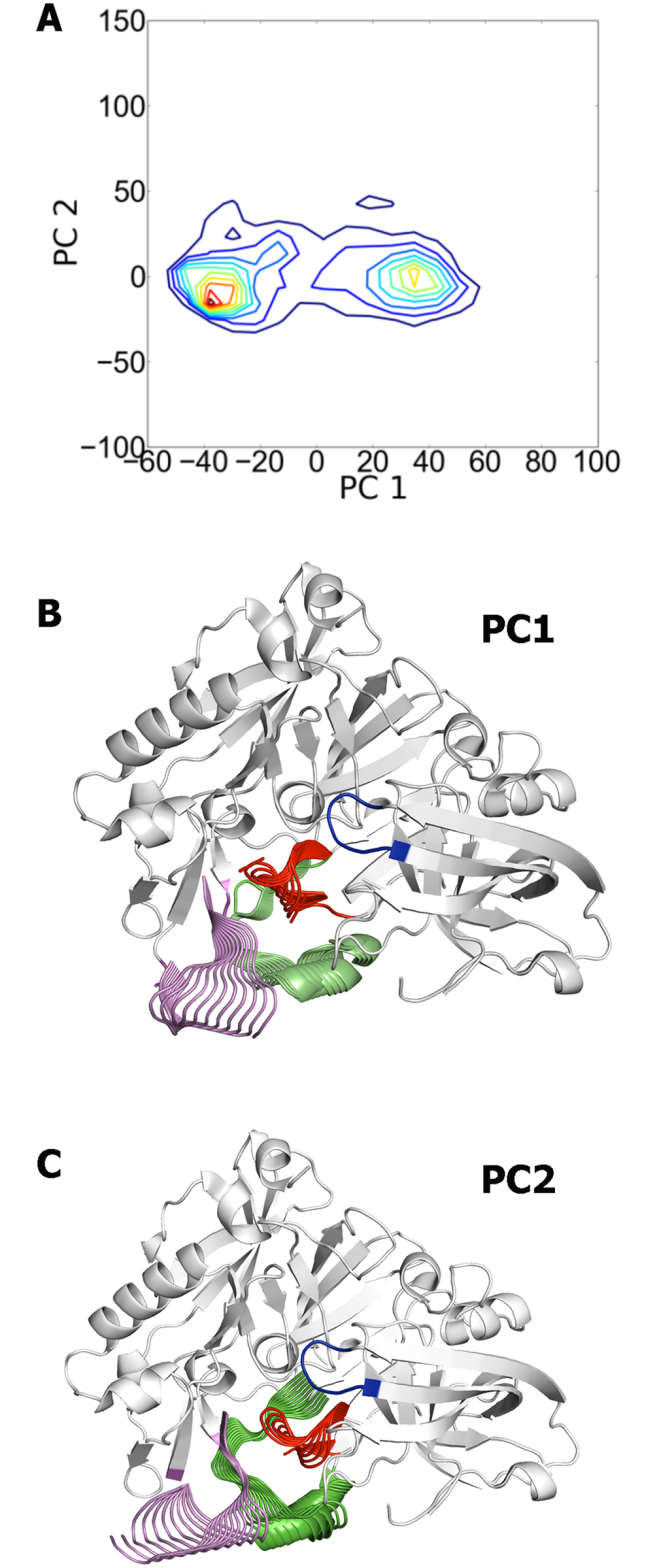
Principal Component Analysis of the MD trajectories. (A) Projection of the ensemble of snapshots collected along the last 50 ns of the 200 MD trajectories on the space defined by the two major principal components (PC1, PC2). (B, C) Representation of the major deformations in loops 8–14, 154–169, and 307–318 generated by the two principal eigenvectors. Deformations are projected onto the average structure of the protein backbone, which is shown as white cartoon with the only exception of the flap, which is shown in blue.

The PCA analysis of the collected frames allowed us to identify the major conformational states of the region shaped by loops 8–14, 154–169, and 307–318. Near 50% of the conformational variance is covered by the first four eigenvectors, which reveals a limited range of conformational flexibility. Indeed, the two first eigenvectors explain around 36% of the structural variance, with contributions of 23% and 13% for the first and second components, respectively. The projection of the sampled snapshots onto the bidimensional map defined by the two principal components reveals the existence of two conformational families (Fig 5), which mostly reflect the *in* and *out* arrangements of the loop 8–14. Noteworthy, it is not just that one cluster comes purely from those simulations begun from the 1SGZ starting structure and the other from those simulations begun from the 2P4J start-point; the significant overlap between the two conformational distributions supports the sampling of structures involved in a smooth transition between the two clusters, and though a rare event it was nevertheless detected in a number of the MD simulations. However, it must be stressed that the structural variance of this region stems from collective fluctuations of the three loops, as noted in the representation of the two principal eigenvectors in Fig 5. Thus, the first eigenvector primarily encodes the correlated motion of loops 8–14 and 307–318, while residues 154–169 retain the helical arrangement found in the X-ray structure. In contrast, a significant feature of the second eigenvector is the loss of the helix and its transition to a disordered loop (Fig 5). Hence, while for the sake of simplicity the labels *in* and *out* will be used hereafter to denote the two conformational clusters, one must keep in mind that the flexibility of the terminal side of the catalytic cleft arises from global fluctuations of the three loops. The contribution of the third and forth components was lower, amounting to 8 and 6%, respectively. Inspection of the plots for the third and forth components showed no relevant additional information (S2 Fig).

### Identification of pocket cavities and docking calculations

The preliminary analysis of the snapshots revealed that loops 8–14, 154–169, and 307–318 are generally tightly packed, but also disclosed the existence of structures characterized by the transient formation of a secondary pocket in this region (S3 Fig), which might be hypothesized to be the binding locus of rhein and chromene moieties in hybrids **1** and **3**, respectively. In order to obtain a better appraisal of the suitability of the secondary pocket for ligand binding, a clustering analysis was conducted separately for the snapshots grouped in the two (*in*, *out*) conformational families shown in Fig 5. Moreover, due to the large sensitivity of the binding capabilities of pockets to the precise geometrical and physicochemical features of side chains, clustering was conducted for the whole set of heavy atoms in loops 8–14, 154–169, and 307–318.

The snapshots pertaining to the *in* conformational state were grouped in 13 clusters. Inspection of representative structures of the first two clusters, which accounted for nearly 87% of the total ensemble, pointed out the presence of a small pocket of around 40–50 Å^3^ (S4 Fig). This volume is unsuitable for binding of the rhein moiety, which has an estimated size of 240 Å^3^. Inspection of the less populated clusters often revealed the presence of slightly larger pockets (around 125 Å^3^; S4 Fig), but again too small to be filled with the rhein moiety.

**Fig 6:**
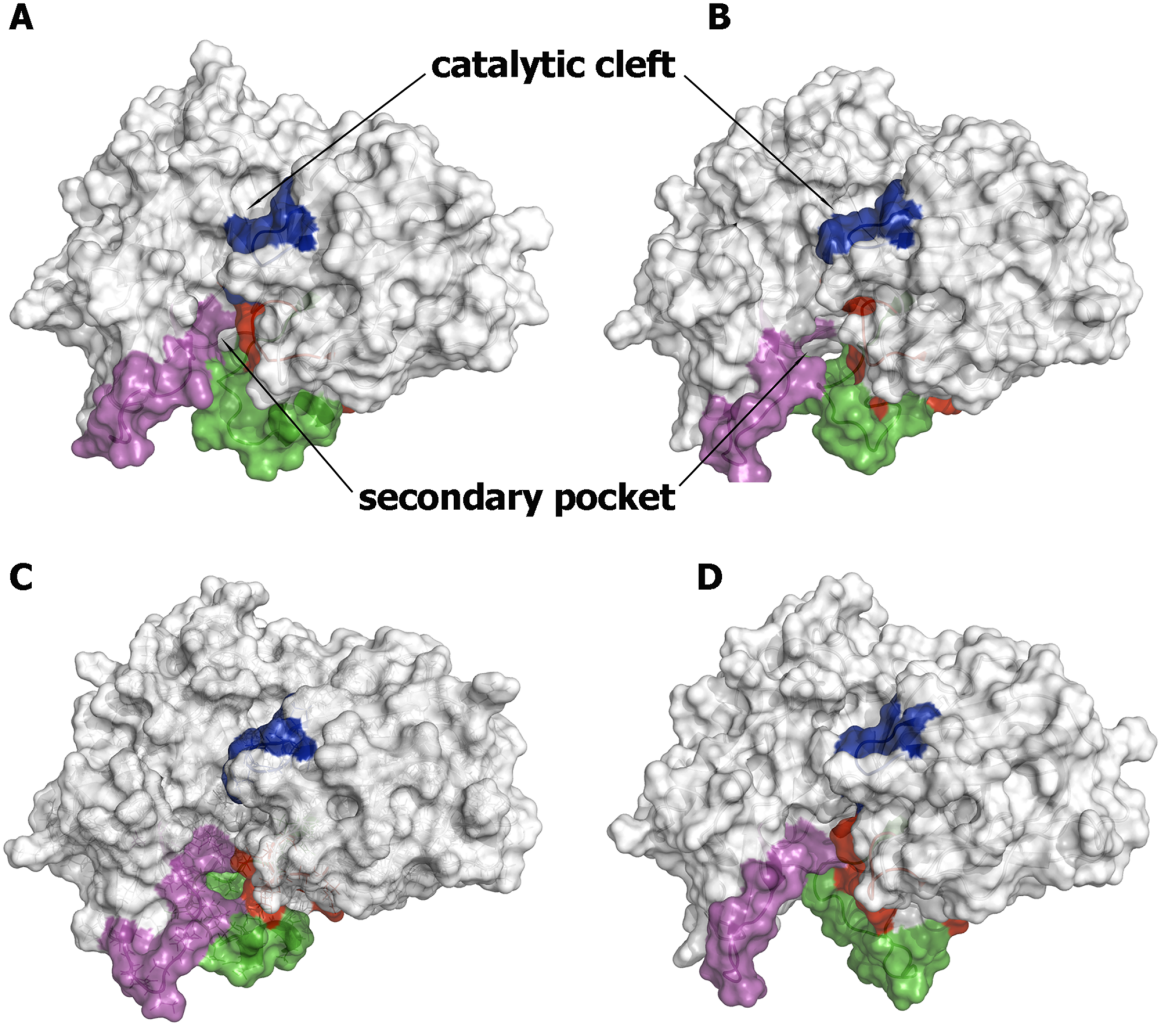
Representative snapshots of BACE-1 with the loop 8–14 in the *out* conformational state. Structures were taken from clusters 13 (1.5%; A), 22 (0.8%; B), 25 (0.5%; C), and 26 (0.4%; D). The flap region is shown in blue, and the loops defined by residues 8–14, 154–169, and 307–318 are shown in red, green and magenta, respectively. The size of the secondary pocket in the selected snapshots is around (A) 460, (B) 520, (C) 770, and (D) 1,250 Å^3^.

In contrast to the preceding findings, up to 50 clusters were found for the structures sampled in the *out* conformational state, thus indicating a larger structural plasticity compared to the *in* conformational family, which is also reflected in the fact that up to 10 clusters are required to achieve 90% coverage of the overall ensemble. Noteworthy, the flexibility of the loops gives rise to the formation of transient pockets covering a wide range of sizes and shapes, often with geometrical features suited for the binding of fragments (Fig 6). Thus, depending on the spatial arrangement of the loops, one can follow the transition from a narrow pocket, with a volume close to 490 Å^3^ (cluster 13; population of 1.5%), to a wide cavity originated from the relative displacement of loops 8–14 and 307–318, leading to a cleft enclosing a volume close to 1,250 Å^3^ (cluster 26; population of 0.4%), which is exceedingly large to be tightly filled with small fragments. Let us note that comparison with X-ray structures is in most cases not feasible due to the lack of precise structural data for the three loops that shape the secondary pocket. However, inspection of the 1SGZ and 2P4J, chosen as representative templates of the *in* and *out* conformational states, respectively (see above), reveals that they have similar pockets, with volumes close to the lowest size (around 50 Å^3^). This can presumably be ascribed to crystal packing effects, while present MD simulations reveal the large conformational plasticity of the loops that shape this floppy pocket.

**Fig 7:**
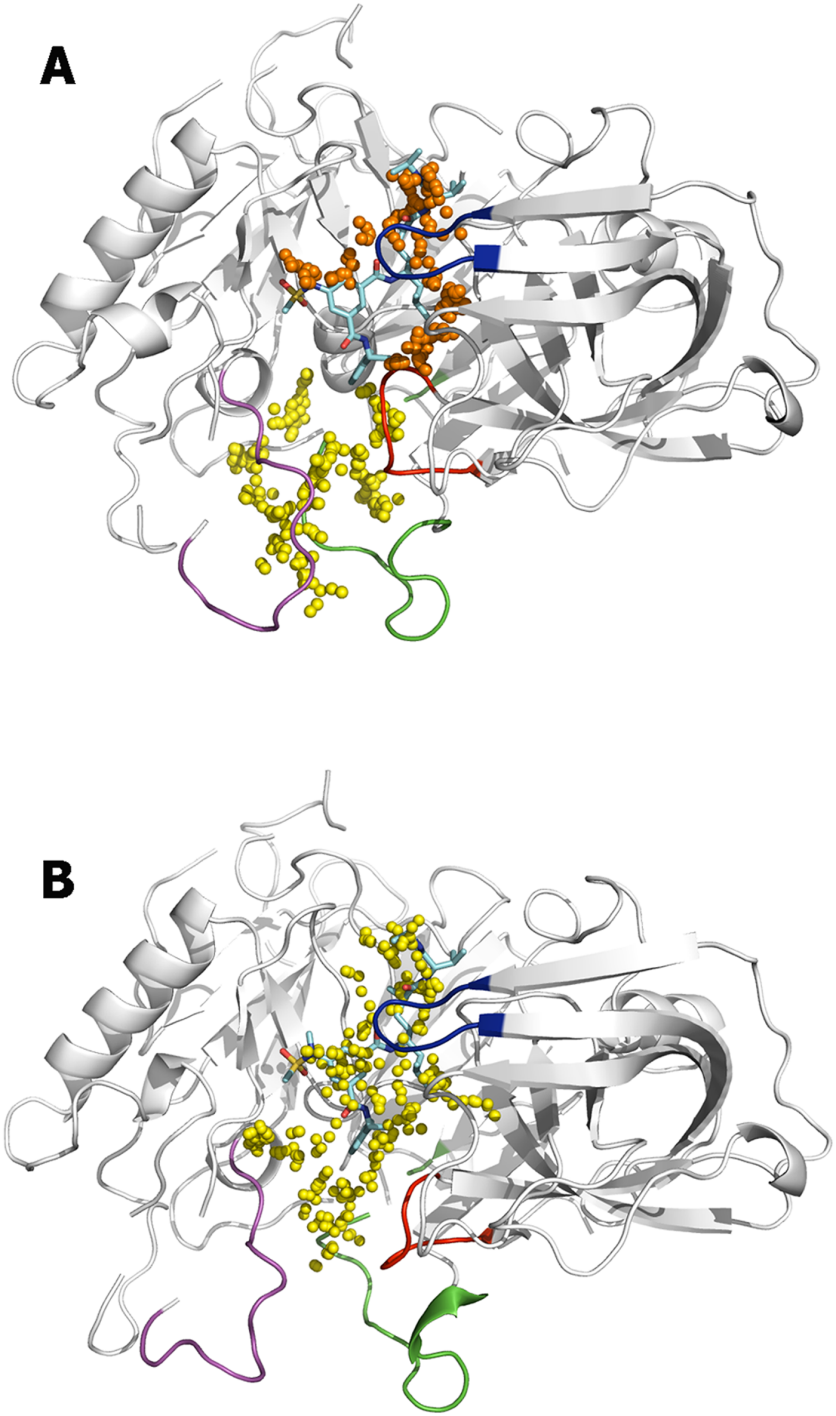
Representation of the druggable sites identified from fPocket analysis. The BACE-1 backbone is shown as white cartoon. (A) The catalytic and secondary pockets found in cluster 13 are shown as orange and yellow spheres. (B) The single pocket found along the catalytic cleft in cluster 21 is shown as yellow spheres. The flap region is shown in blue, and the loops defined by residues 8–14, 154–169, and 307–318 are shown in red, green and magenta, respectively. The ligand located along the catalytic cleft in structure 2P4J is shown as cyan-colored sticks.

Overall, the preceding analysis reveals that the formation of the secondary pocket cannot be simply ascribed to the conformational change of a specific residue, as has been described in other cases. For instance, the accessibility to cryptic pockets at the protein-binding interface of eIF4E depends on the gating movement of Trp73 [57], or the disclosure of a hydrophobic pocket by side-chain movements of Tyr417 and Tyr481 in polo-like kinase 1 [58]. Rather, the collective motions of loops 8–14, 154–169, and 307–318 determine the structural features of the cryptic pocket found in BACE-1, which exhibits a large variation in the overall shape and size (Figs 5 and 6). It is then reasonable to expect that the plasticity of these structural elements will affect the ability of the secondary pocket to interact with small molecules. The linkage between conformational plasticity and ligandability of the secondary pocket has been explored by using the fPocket program, which permits to estimate the druggability of the distinct clustered pockets. To this end, fPocket provides a druggability score (comprised between 0 and 1) based on selected physicochemical features of the cavity [45]. Thus, pockets limited to the region confined by the three loops exhibit low druggability (typically less than 0.1). Only structures that allow the extension of the secondary pocket toward the catalytic site lead to enhanced druggability. This is noted through the comparison of the pockets found along the catalytic cleft in clusters 13 and 21, where the gross structural features of the binding pocket exhibit a large resemblance and have a druggability score of 0.52 and 0.70 (Fig 7). The former reveals the extension of the secondary cavity toward the S_4_ subpocket, which is filled by a benzene ring attached to the isophthalamide moiety of the inhibitor bound to BACE-1 in structure 2P4J (Fig 7A). Nevertheless, there is no direct connection with the pocket found around the catalytic dyad, which accommodates well the isophthalamide scaffold. In this case, the druggability of the catalytic pocket is around 2-fold larger relative to the secondary pocket (0.35 vs. 0.17, respectively). At large extent, this can be ascribed to the different balance between hydrophobic/hydrophilic areas in the catalytic and secondary pockets. Albeit the total volume and surface area of the two pockets is similar (1150–1250 Å^3^, and 640–660 Å^2^), the catalytic pocket is primarily hydrophobic (around 68% of the accessible surface, due to the presence of residues Leu30, Tyr71, Phe108, Trp115, Ile118, Tyr198 and Ile226; see below), but the reverse trend is found for the secondary pocket, due to the presence of polar residues such as Lys10, Ser11, Glu311, and specially Arg403 (see below). By contrast, the results obtained for cluster 21 (population of 1.0%) reveal that the secondary cavity is fused with the active site forming a single cavity along the catalytic cleft (Fig 7B). Importantly, the distance between the centroids of the catalytic and secondary sites is close to 13 Å, thus satisfying the geometrical criteria required for the attachment of both huprine and rhein moieties through the nonamethylenic tether in hybrid **1**.

As a final test, docking calculations were performed to check the feasibility of the pockets to bind the rhein unit. At this point, let us note that the huprine moiety is assumed to bind the primary catalytic pocket, since it should have a net positive charge (the pK_a_ of huprine Y has been determined to be close to 8.9 [59]). It is then reasonable to expect that it will form an electrostatically favored interaction with the negative charge of the Asp dyad. On the other, an electrostatic repulsion may be expected upon binding to the cryptic pocket due to the presence of Arg307 (see below). Accordingly, the rhein moiety should be the fragment that binds this latter binding site. The results reveal the similar arrangement found for the best pose of rhein in clusters 13 and 21 (Fig 7). With regard to cluster 13, 10 out of the first 13 best scored poses shared the arrangement shown in Fig 7A, with a score of -22 kcal/mol, whereas for cluster 21 the first 24 poses were ranked with a scoring comprised between -22 and -21 kcal/mol (Fig 7B). In all cases, a common feature is the interaction between the guanidinium moiety of Arg307 and the carbonyl group of the most hydrophobic edge of rhein, suggesting that this residue can be also relevant for the binding of the chromene unit present in hybrid **3**, while the most hydrophilic edge of the rhein moiety is oriented toward the bulk solvent.

### Molecular dynamics simulations of the holo enzyme

**Fig 8:**
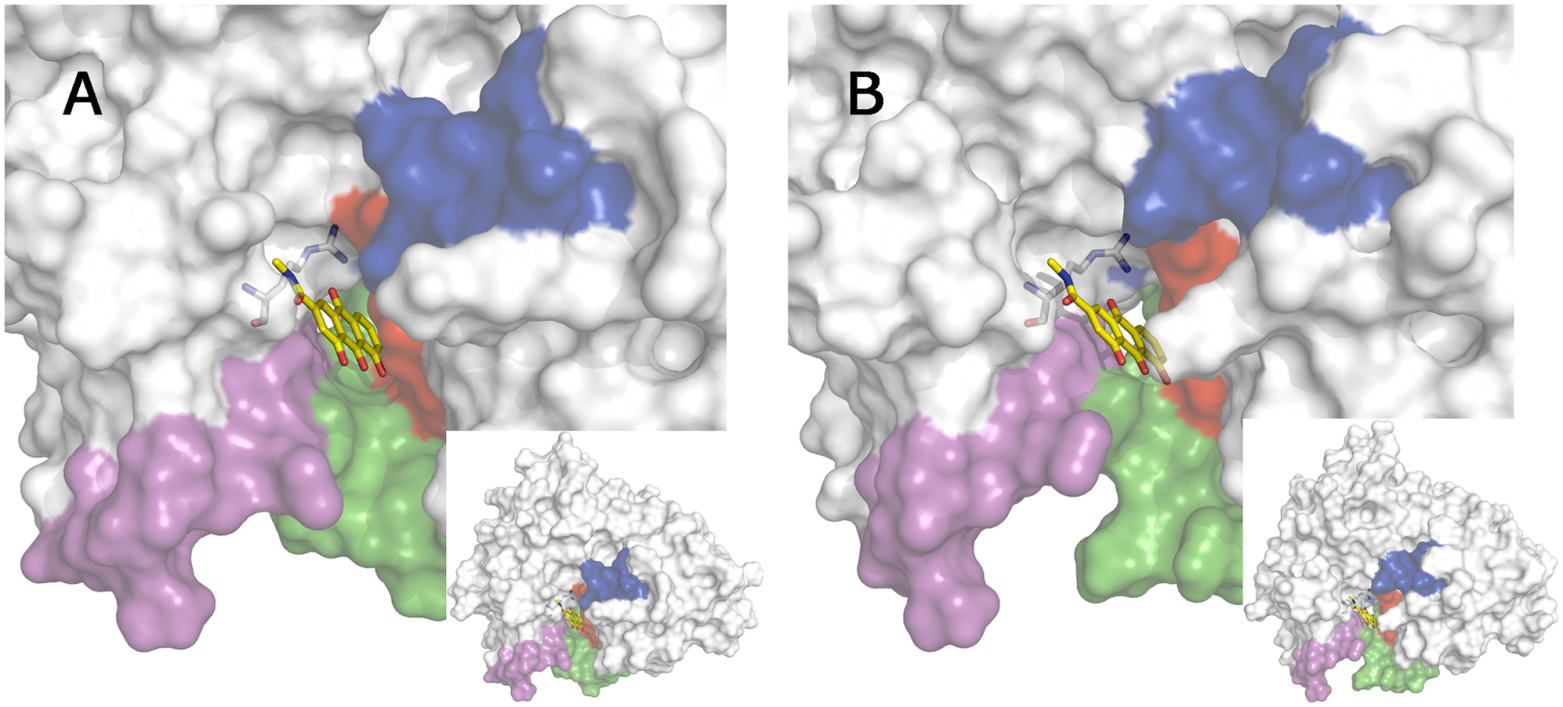
Surface representation of the best pose obtained for the docking of rhein to the secondary pocket in clusters (A) 13 and (B) 21. The BACE-1 backbone is shown as white cartoon. The flap region is shown in blue, and the loops defined by residues 8–14, 154–169, and 307–318 are shown in red, green, and magenta, respectively. Arg307 is shown as sticks. The inset depicts the location of the binding pocket in the whole protein.

The structural integrity of the binding mode of rhein was examined by means of MD simulations run for the BACE-1 complexes with hybrid compounds **1** and **3**. To this end, six independent simulations were run for (+)-**1**, (–)-**1**, and **3**, which were aligned along the cleft using the best docked pose for the rhein moiety (Fig 8) and the binding mode of the huprine moiety found in previous MD simulations of the BACE-1 complex with hybrid **2** [29]. For each compound, two distinct arrangements were adopted for the amide group attached to the rhein/chromene unit, so that 3 MD simulations were run with the carbonyl group oriented toward Arg307 or in the opposite way.

**Fig 9:**
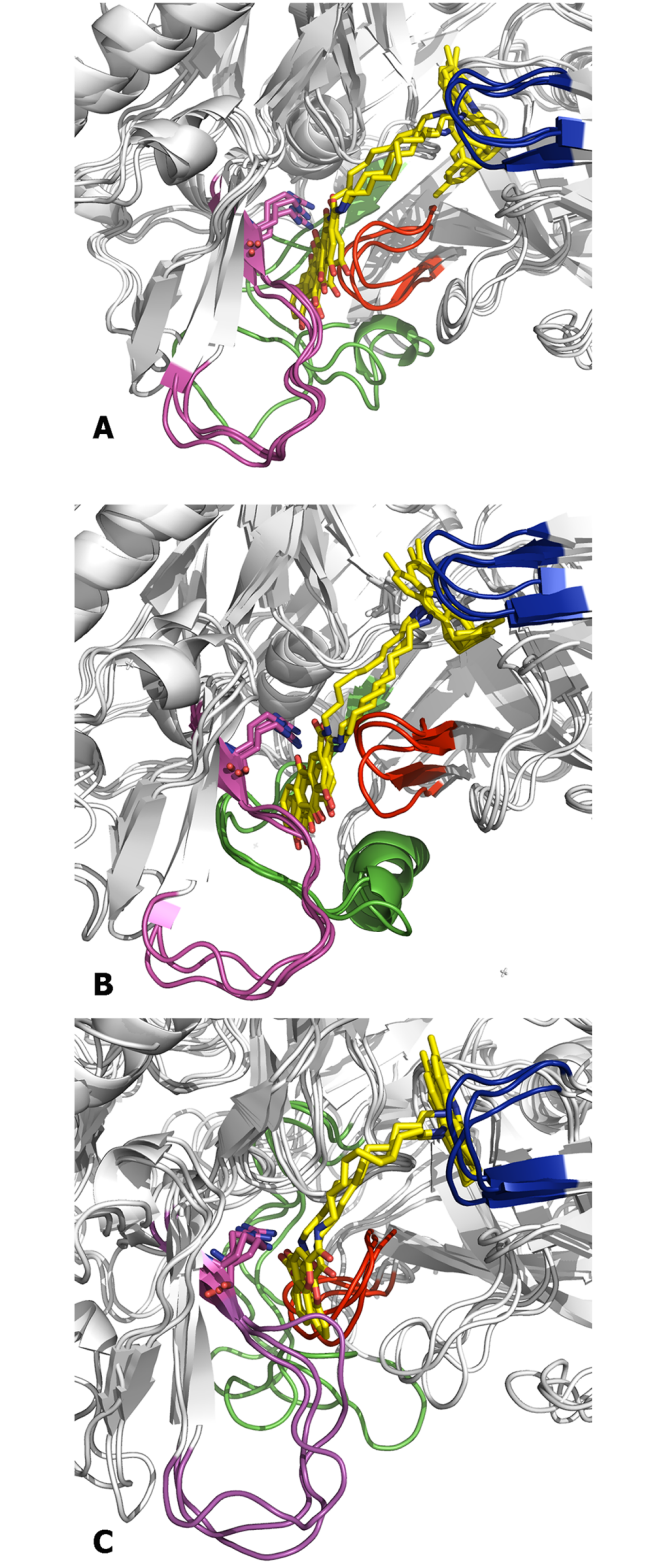
Superposition of the snapshots collected at the end of the trajectories run for the BACE-1 complexes with hybrid compounds (A) (+)-1, (B) (–)-1, and (C) 3. The protein backbone is shown as white cartoon. The flap region is shown in blue, and the loops defined by residues 8–14, 154–169, and 307–318 are shown in red, green and magenta, respectively. Arg307 is shown in sticks.

The results pointed out that the hybrid compound **1** remained firmly bound to the enzyme along the six independent simulations (Figs 9A and 9B). The huprine moiety was tightly bound to the active site in all cases. This can be attributed to the strong electrostatic stabilization arising from the hydrogen-bond interaction formed between the protonated aminoquinoline system and the catalytic dyad (Asp32), as noted in an average distance (from the protonated N atom of huprine to the carboxylate oxygen of Asp32) of 2.89 ± 0.14 Å (S5 Fig). Furthermore, the aminoquinoline system of the huprine moiety was found to fill the hydrophobic pocket formed by residues Leu30, Tyr71, Phe108, Trp115, and Ile118, forming van der Waals contacts typically ranging from 3.9 to 4.5 Å. Finally, the chlorine atom filled a subpocket formed by the side chains of Tyr198 and Ile226 (van der Waals contacts, on average, of 4.0 ± 0.32 and 4.15 ± 0.56 Å, respectively). In particular, both enantiomeric forms of the huprine unit were well accommodated under the flap.

On the other hand, the hydroxyanthraquinone moiety adopted a well-defined binding mode in the secondary cavity (S6 Fig). The most relevant feature was the hydrogen bond interaction of the carbonyl unit of rhein with the guanidinium moiety of Arg307 (average distance of 2.96 ± 0.22 Å, which in turn formed a salt bridge interaction with Glu339. Furthermore, the carbonyl group located on the edge of the rhein moiety opposite to Arg307 formed an electrostatic stabilization with the main chain NH group of Glu310. Finally, the stability of this arrangement was enhanced by the interaction between the side chains of Glu310 and Ser10, thus forming a hydrogen-bond (average distance of 2.99 ± 0.82 Å; see S6A Fig) staple that favoured the binding of the rhein moiety to the secondary pocket (found in around 63% of the ensemble of snapshots collected in the last 20 ns of the MD simulations). Less frequently, such staple interaction involved the formation of a salt bridge between Glu310 and the protonated amino group of Lys9 (around 9%; S6B Fig), and eventually a triple motif formed by the hydrogen bond between Glu311 and Ser10 in conjunction with the salt bridge between Glu310 and Lys9 was also observed (4%). On the other hand, it is worth noting that the pattern of interactions described above was not affected by the specific arrangement imposed to the amide group in the tether, and the major interactions formed between the inhibitor and the enzyme were similar in the six simulations run for (+)-**1** and (–)-**1**.

Compared to the binding of compound **2** to the enzyme cleft, which was examined by docking and molecular dynamics simulations in a previous work [29], compounds **1** and **2** exhibit a similar overall alignment along the substrate binding cleft. However, the larger length of the tether in compound **1**, and the increased flexibility of the methylenic chain, permits a better accommodation of the rhein moiety at the secondary site, particularly enabling the interaction with the guanidinium moiety of Arg307. In contrast, this interaction was not stably observed in the molecular modeling studies reported for the binding of compound **2** [29]. Hence, this would explain the 80-fold increase in inhibitory potency upon replacement of the aromatic tether present in **2** by the nonamethylene linker in **1**.

Noteworthy, a similar arrangement was found for the tacrine-chromene hybrid **3**, whose chromene carbonyl group is also capable of forming electrostatic interactions with Arg307 (Fig 9C). Hence, our results suggest that both rhein and chromene moieties can be viewed as bioisoteric groups as they share similar interactions with the secondary pocket. It is also worth noting that the oligomethylenic tether of hybrids **1** and **3** adopts a similar arrangement in the substrate binding pocket, which enables both chromene and rhein moieties to fill similar regions in the secondary pocket.

The binding model shown in Fig 9 allows us to rationalize the structure-activity relationships observed for the two hybrid compounds. First, regarding the huprine-rhein hybrid, the results reported in ref. 29 showed a notable dependence of the inhibitory activity on the length of the tether. This trend can be easily explained by the expected alteration in the arrangement of the rhein unit, which would affect the electrostatic stabilization with Arg307. Thus, lengthening or shortening of the tether from a nonamethylenic chain by a single methylene unit changes the inhibitory potency from an IC_50_ value of 80 nM to around 1 μM. Furthermore, the fact that huprine-rhein hybrids with pentamethylene to heptamethylene tethers proved to be inactive for BACE-1 inhibition at a concentration of 1 μM [29] can be explained by the absence of a suitable binding for the rhein moiety along the enzyme cleft (data not shown). On the other hand, the identical IC_50_ value obtained for both (+)-**1** and (–)-**1** suggests that the two enantiomeric forms of the huprine moiety are well arranged at the catalytic site, while their binding should facilitate the proper accommodation of the rhein moiety to the secondary site at the terminus of the catalytic cleft.

With regard to the tacrine-chromene hybrid **3**, the dependence of the tether length on the inhibitory activity was not explicitly reported in the work by Fernández-Bachiller et al. [30]. The present results suggest that these hybrids should exhibit a similar dependence as that found for the huprine-rhein hybrids. Nevertheless, the authors checked the effect of different chemical modifications in positions 5’, 6’, and 7’ of the chromene ring, to find that the inhibitory activity was nearly unaffected by changes involving different substitution patterns with hydroxy and methoxy groups in these positions (*K*_*i*_ values ranging from 0.62 to 3.99 μM). The lack of sensitivity to these changes can be ascribed to the solvent exposure of this part of the chromene ring in the binding mode reported in the present MD simulations, which would not participate directly in contacts with the secondary pocket nor would affect the electrostatic interaction of the carbonyl group with Arg307.

## Conclusion

The results presented in this study support the reliability of combining extended MD simulations with the analysis of collective dynamics to disclose “floppy” pockets hidden in highly flexible regions and to exploit these secondary pockets for the design of novel drugs hitting cryptic pockets in challenging targets [60–62], such as BACE-1. Within the plethora of the therapeutically useful proteins codified by the “druggable genome”, *β*-secretase (BACE-1) has been classified in the subset of the target receptors considered of “difficult” ligandability through small molecules [63]. Hence, the identification of novel sites that could provide additional anchoring points might be highly valuable for increasing the chemical diversity of drug candidates, enhancing the likelihood of finding suitable scaffolds for the design of effective BACE-1 inhibitors.

In this context, this study supports the feasibility of formation of transient secondary pockets in the region shaped by the highly flexible loops 8–14, 154–169, and 307–318. The plasticity of these loops is revealed by the formation of pockets that cover a wide range of sizes and shapes, some with druggability features that would permit the binding of small fragments, such as the rhein and chromene moieties of hybrids **1** and **3**. Importantly, the results provide a basis to rationalize the trends in inhibitory potency observed for the huprine-rhein and tacrine-chromene hybrids, particularly the large dependence of the inhibitory potency on the tether length observed in the former and the insensitivity to the change of substituents in the chromene moiety of the latter. Finally, the disclosure of this druggable, “floppy” pocket paves the way for future studies focused on virtual screening of fragments that can stabilize the open state of this cryptic pocket, enabling the structure-guided design of novel BACE-1 inhibitors.

## Supporting information

S1 Fig Represention and numbering of the subsites in the substrate binding cleft of BACE-1. The enzyme is shown as a white surface using the X-ray structure of the complex with a substrate mimetic inhibitor (with carbon atoms shown as green-colored sticks) (PDB entry 1XN3). The flap region is shown in deep blue.
(TIF)

S2 Fig Principal Component Analysis of the MD trajectories. Projection of the ensemble of snapshots collected along the last 50 ns of the 200 MD trajectories on the space defined by (A) the first and third principal components (PC1, PC3), and (B) the second and third principal components (PC2, PC3).
(TIF)

S3 Fig Representative snapshots of BACE-1 taken from the MD conformational ensemble. The two major clusters denote structures characterized by the loop 8–14 in both *in* (A, C) and *out* (B, D) conformations. The flap region is shown in blue, and the loops defined by residues 8–14, 154–169, and 307–318 are shown in red, green and magenta, respectively. While generally the loops are tightly packed, a secondary pocket is transiently formed as shown for representative structures of the enzyme (C, D).
(TIF)

S4 Fig Representative snapshots of BACE-1 with the loop 8–14 in the *in* conformational state. Structures were taken from to clusters 1 (75%; A) and 10 (<1%; B). The flap region is shown in blue, and the loops defined by residues 8–14, 154–169, and 307–318 are shown in red, green and magenta, respectively. A small secondary pocket of (A) 38 and (B) 127 Å^3^ is displayed in the two structures.
(TIF)

S5 Fig Representative view of the binding mode of the huprine moiety of compound 1 to the catalytic site of BACE-1. The protein backbone is shown as white cartoon, but for the residues in the flap loop, which are shown in blue. Selected residues in the binding pocket are shown as sticks. Plots A and B display the two enantiomeric forms of the huprine moiety, which is shown with carbon atoms colored in yellow. For the sake of clarity, the methylenic chain and the rhein moiety have been deleted. Likewise, only polar atoms in the huprine moiety and Asp228 are shown.
(TIF)

S6 Fig Representative views of the binding mode of the rhein moiety of compound 1 to the secondary site of BACE-1. The protein backbone is shown as white cartoon, but for residues in the loops 8–14, 154–169, and 307–318, which are shown in red, green, and magenta, respectively. Selected residues in the binding pocket are shown as sticks. Plots A-C display distinct arrangements of residues Lys9, Ser10, and Glu310. For the sake of clarity, the methylene chain and the huprine moiety have been deleted. Only selected polar atoms are shown.
(TIF)

S1 Table Compilation of MD simulations reported in this study for the apo and holo forms of the BACE-1. (PDF)
