# Correction: Unveiling a novel transient druggable pocket in BACE-1 through molecular simulations: Conformational analysis and binding mode of multisite inhibitors

**DOI:** 10.1371/journal.pone.0190327

**Published:** 2017-12-21

**Authors:** Ornella Di Pietro, Jordi Juárez-Jiménez, Diego Muñoz-Torrero, Charles A. Laughton, F. Javier Luque

There is an error in the Funding section. The correct Funding statement is as follows: This work was supported by the Ministerio de Economía y Competitividad (SAF2014-57094-R), Generalitat de Catalunya (GC; 2014SGR1189, 2014SGR52), and ICREA Academia (FJL). The Consorci de Serveis Universitaris de Catalunya (CSUC; FJL) is acknowledged for providing computational resources. This work used the ARCHER UK National Supercomputing Service (http://www.archer.ac.uk), via the HECBioSim consortium (EPSRC Grant EP/L000253/1), and the Spanish Barcelona Supercomputer Center (https://www.bsc.es; project BCV-2017-2-0012). A fellowship from GC to ODP is gratefully acknowledged. The funders had no role in study design, data collection and analysis, decision to publish, or preparation of the manuscript.
