# Supplementary figures and images for: Unveiling a novel transient druggable pocket in BACE-1 through molecular simulations: Conformational analysis and binding mode of multisite inhibitors
Source: PLoS One. 2017 May 15;12(5):e0177683. doi: 10.1371/journal.pone.0177683 (PMC5432175; doi:10.1371/journal.pone.0177683)

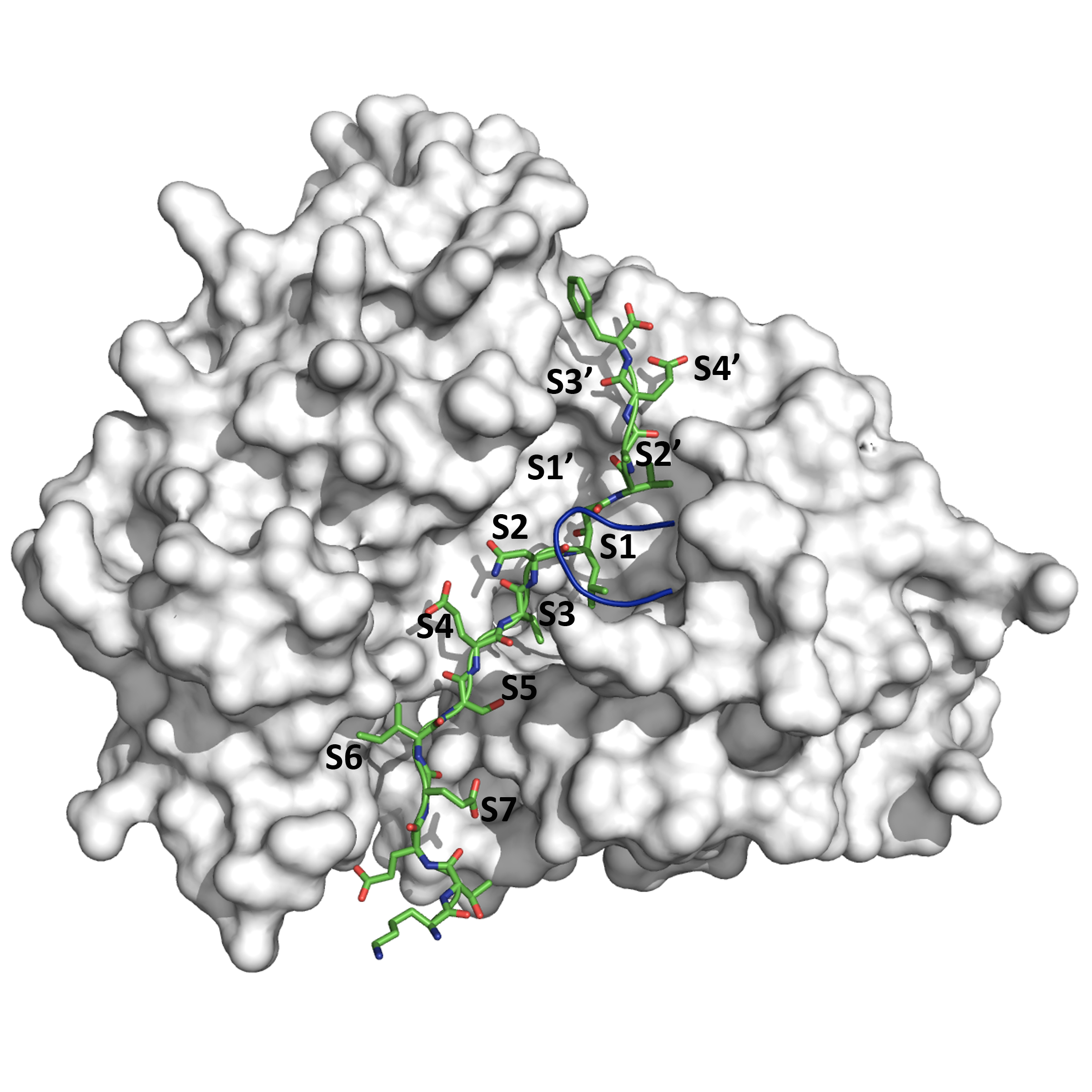

Supplement: S1 Fig — The enzyme is shown as a white surface using the X-ray structure of the complex with a substrate mimetic inhibitor (with carbon atoms shown as green-colored sticks) (PDB entry 1XN3). The flap region is shown in deep blue. (TIF) [file pone.0177683.s001.tif]

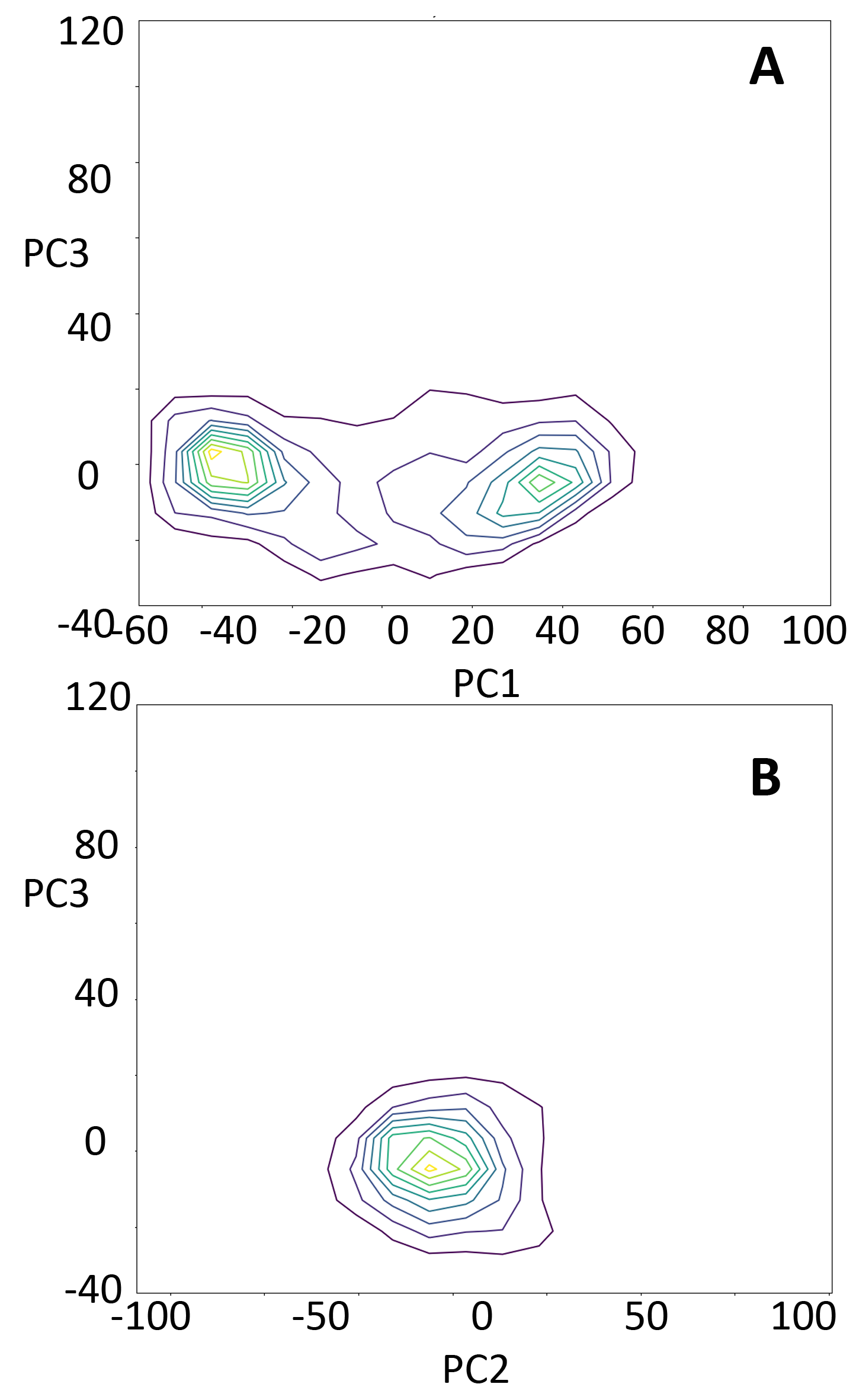

Supplement: S2 Fig — Projection of the ensemble of snapshots collected along the last 50 ns of the 200 MD trajectories on the space defined by (A) the first and third principal components (PC1, PC3), and (B) the second and third principal components (PC2, PC3). (TIF) [file pone.0177683.s002.tif]

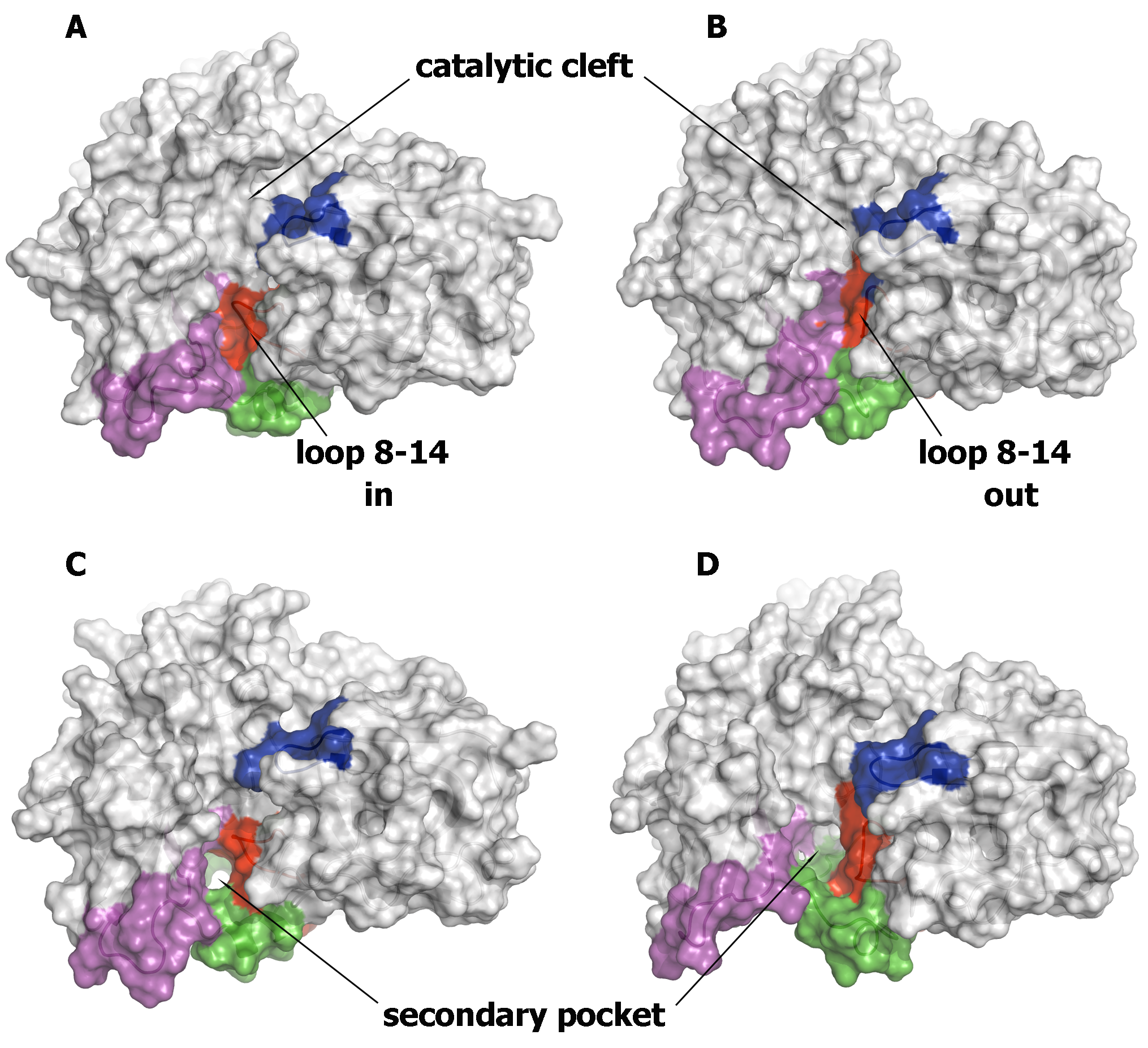

Supplement: S3 Fig — The two major clusters denote structures characterized by the loop 8–14 in both in (A, C) and out (B, D) conformations. The flap region is shown in blue, and the loops defined by residues 8–14, 154–169, and 307–318 are shown in red, green and magenta, respectively. While generally the loops are tightly packed, a secondary pocket is transiently formed as shown for representative structures of the enzyme (C, D). (TIF) [file pone.0177683.s003.tif]

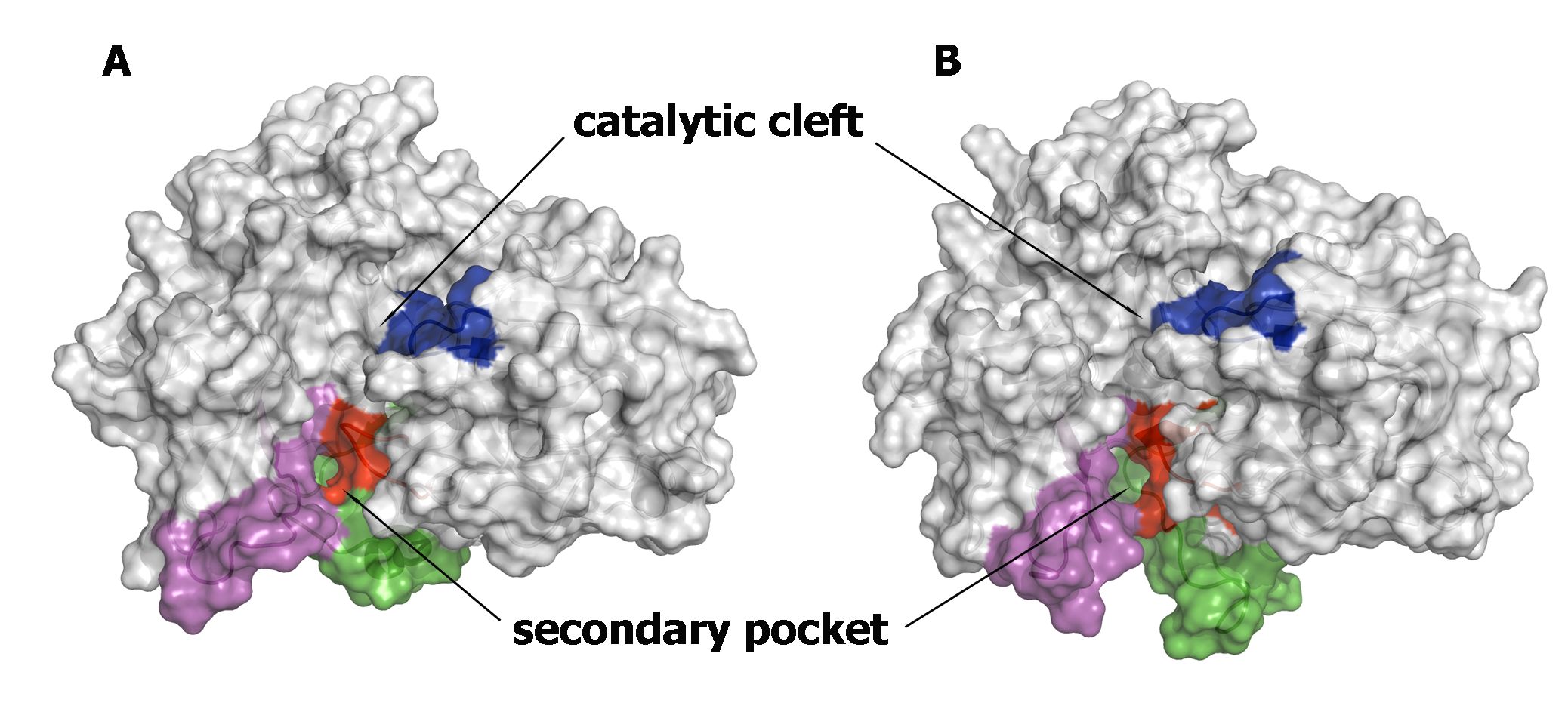

Supplement: S4 Fig — Structures were taken from to clusters 1 (75%; A) and 10 (<1%; B). The flap region is shown in blue, and the loops defined by residues 8–14, 154–169, and 307–318 are shown in red, green and magenta, respectively. A small secondary pocket of (A) 38 and (B) 127 Å3 is displayed in the two structures. (TIF) [file pone.0177683.s004.tif]

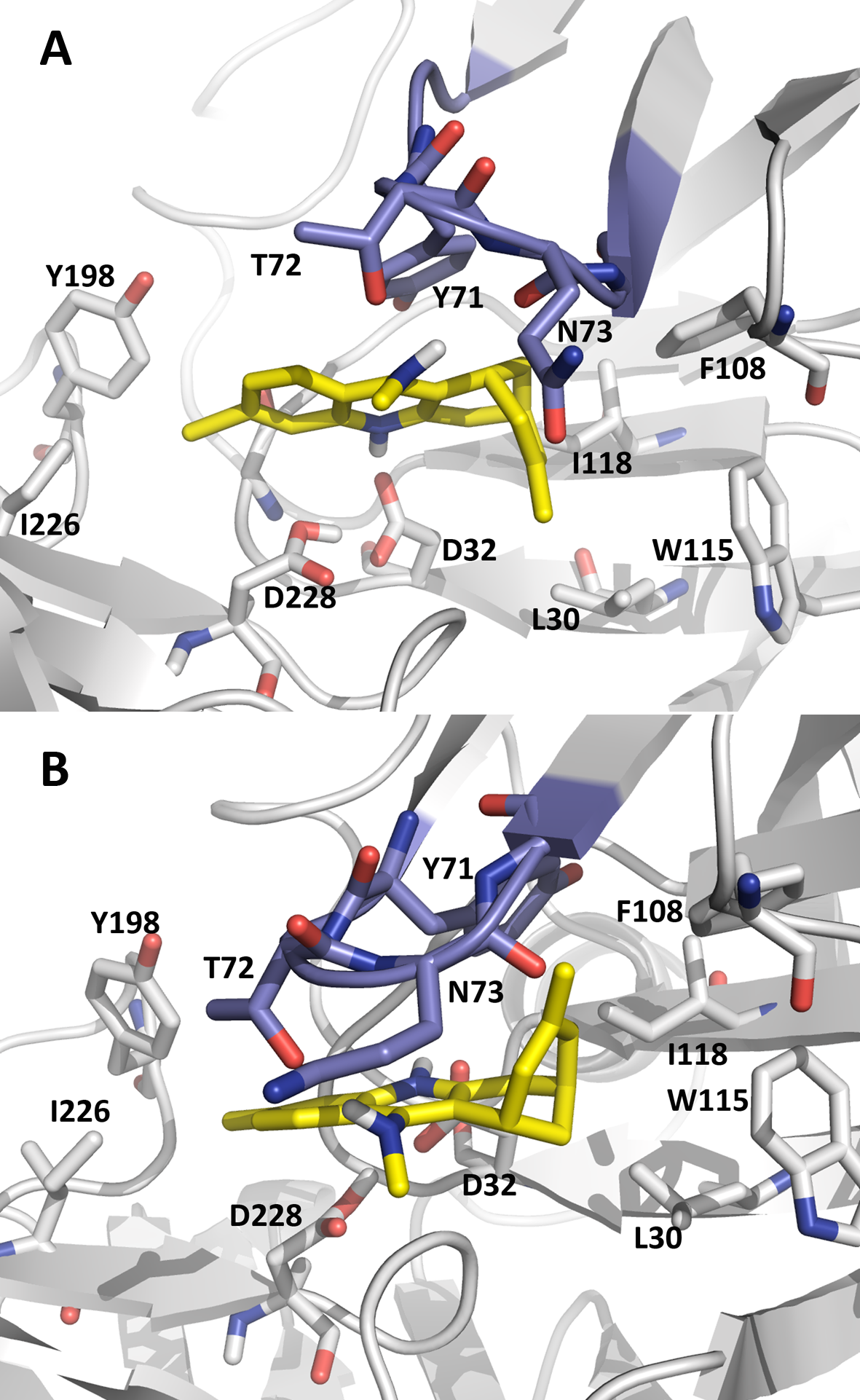

Supplement: S5 Fig — The protein backbone is shown as white cartoon, but for the residues in the flap loop, which are shown in blue. Selected residues in the binding pocket are shown as sticks. Plots A and B display the two enantiomeric forms of the huprine moiety, which is shown with carbon atoms colored in yellow. For the sake of clarity, the methylenic chain and the rhein moiety have been deleted. Likewise, only polar atoms in the huprine moiety and Asp228 are shown. (TIF) [file pone.0177683.s005.tif]

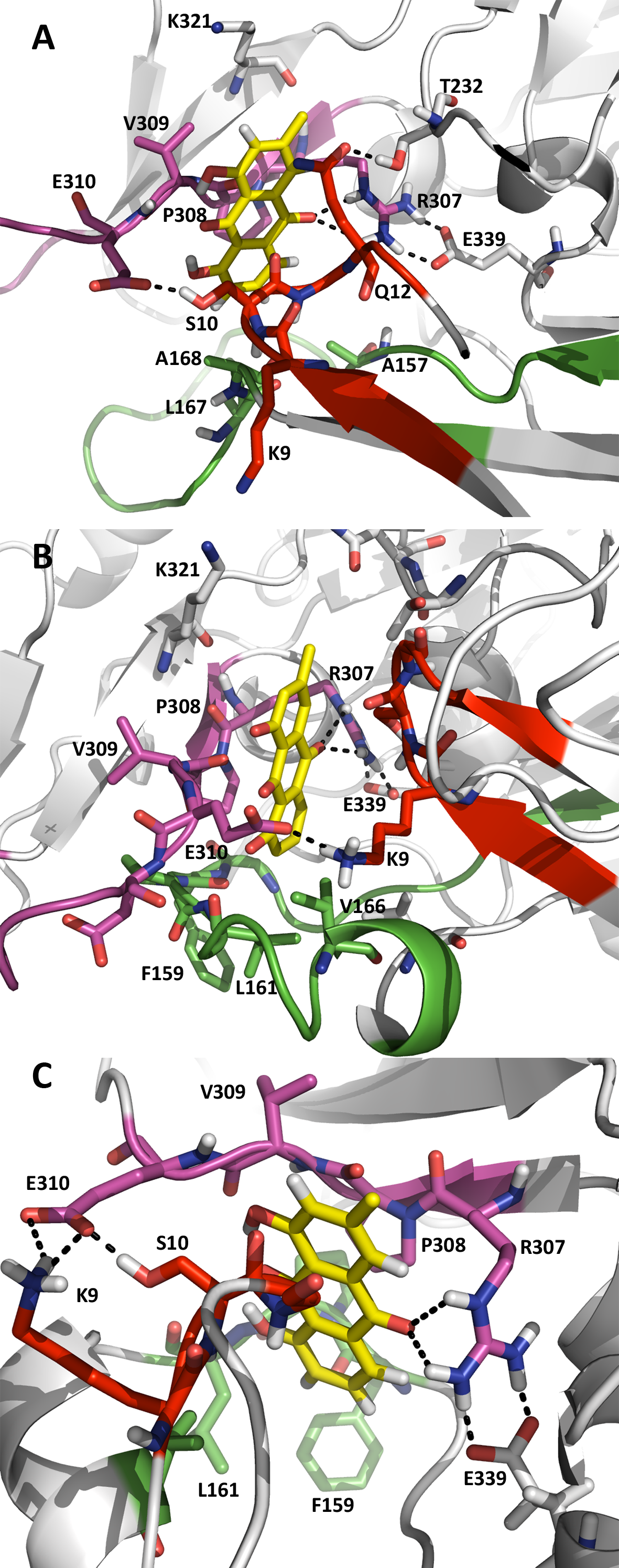

Supplement: S6 Fig — The protein backbone is shown as white cartoon, but for residues in the loops 8–14, 154–169, and 307–318, which are shown in red, green, and magenta, respectively. Selected residues in the binding pocket are shown as sticks. Plots A-C display distinct arrangements of residues Lys9, Ser10, and Glu310. For the sake of clarity, the methylene chain and the huprine moiety have been deleted. Only selected polar atoms are shown. (TIF) [file pone.0177683.s006.tif]
