# Supplementary material for: Unveiling a novel transient druggable pocket in BACE-1 through molecular simulations: Conformational analysis and binding mode of multisite inhibitors
Source: PLoS One. 2017 May 15;12(5):e0177683. doi: 10.1371/journal.pone.0177683 (PMC5432175; doi:10.1371/journal.pone.0177683)
Supplement: S1 Table — (PDF) [file pone.0177683.s007.pdf]

	MD	REPLICAS	TIME (ns)	ANALYSIS
APO	Loop-IN (PDB ID: 1sgz)	100	(100) X 100	**Principal Component Analysis**<br>1st: 23%, 2nd: 13%, 3d: 8%<br>**Clustering**<br>Loop-IN: 13 clusters, with pocket sizes from 40 to 125 Å^3<br>Loop-OUT: 50 clusters; pocket sizes from 490 to 1,250 Å^3<br>**Druggability**<br>Cluster 13 (population of 1.5%): 0,35 (catalytic site), 0.17 (secondary site)<br>Cluster 21 (population of 1.0%): 0.70
	Loop-OUT (PDB ID: 2p4j)	100	(100) X 100	
HOLO	(+)-1 [conformer A]	3	(3) X 50	**Binding mode**<br><br>Catalytic site:<br>Asp32, Asp228<br>Leu30, Tyr71, Phe108, Trp115, and Ile118, Tyr198, Ile226<br><br>Secondary site:<br>Arg307-Glu339<br>Lys 9, Ser10,Glu310<br>Phe159, Leu161, Leu 167, Pro308, Val309
	(−)-1 [conformer A]	3	(3) X 50	
	3 [conformer A]	3	(3) X 50	
	(+)-1 [conformer B]	3	(3) X 50	
	(−)-1 [conformer B]	3	(3) X 50	
	3 [conformer B]	3	(3) X 50	
